# Aggressive Extraocular Sebaceous Carcinoma Recurring after Mohs Micrographic Surgery

**DOI:** 10.1155/2015/534176

**Published:** 2015-01-22

**Authors:** Konstantin V. Grigoryan, Laurel Leithauser, Hugh M. Gloster

**Affiliations:** ^1^University of Cincinnati College of Medicine, Cincinnati, OH 45267, USA; ^2^Department of Dermatology, University of Cincinnati College of Medicine, Cincinnati, OH 45267, USA

## Abstract

Sebaceous carcinomas (SC) are rare adnexal tumors with possible aggressive behavior usually arising in the head and neck region of adults in the seventh decade of life. Treatment has traditionally been with surgical excision with 5-6 mm wide margins but Mohs micrographic surgery (MMS) has also been reported as an effective treatment modality. We present a case of a Caucasian female renal transplant patient with a rapidly enlarging nodule on the left preauricular cheek that was excised with MMS with negative margins. The tumor recurred rapidly and metastasized ultimately leading to the death of the patient. There was some disagreement amongst pathologists as to the possible nature of the diagnosis with the original biopsy being labeled as a poorly differentiated carcinoma. We aim to highlight the potential aggressive nature of SC and review the features of the neoplasm including histological features that help in making the diagnosis.

## 1. Introduction

Extraocular sebaceous carcinoma (SC), a rare malignant tumor usually arising in the head and neck region, may be aggressive with the potential for nodal and distant metastases. Traditionally, treatment consists of surgical excision with 5-6 mm margins. However, recent reports have concluded that Mohs micrographic surgery (MMS) is a viable alternative with few recurrences [1, 2]. Here we present a patient with SC who was treated with MMS with negative margins but had recurrence at the excision site and metastasis shortly after treatment.

## 2. Case Report

**Figure 1:**
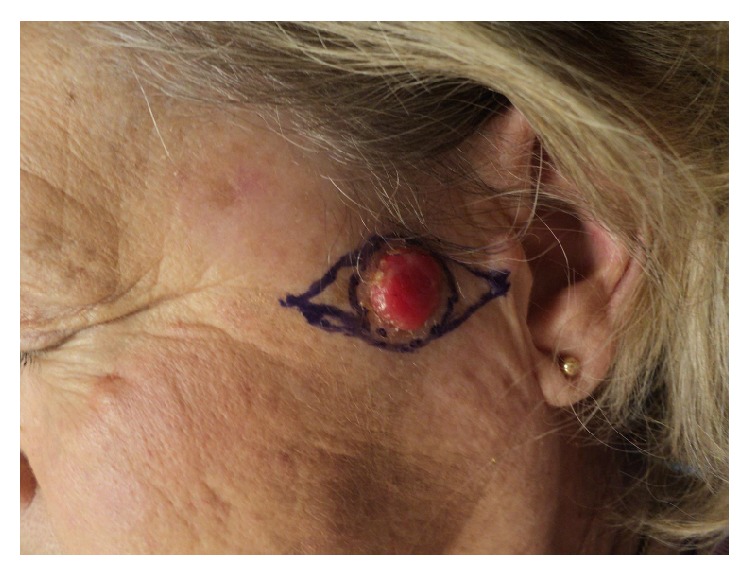
Original patient presentation with preauricular nodule.

A 65-year-old Caucasian woman presented for evaluation of a rapidly enlarging nodule on the left preauricular cheek. The patient had diabetes mellitus type II and hypertension and was on immunosuppressive therapy for renal transplantation 8 years prior. Two years prior she had a cutaneous squamous cell carcinoma in situ (SCCis) on her left third finger that was excised using MMS. The patient was otherwise healthy on presentation. Physical examination revealed a 1.5 cm eroded erythematous nodule on the left preauricular cheek (Figure 1). Initial shave biopsy showed an ulcerated neoplasm within the whole dermis extending to all margins of the specimen consisting of multiple irregular islands of atypical cells that stained uniformly with antibodies to pan keratin and uniformly negative with antibodies to S100 protein. A diagnosis of a poorly differentiated carcinoma was made.

**Figure 2:**
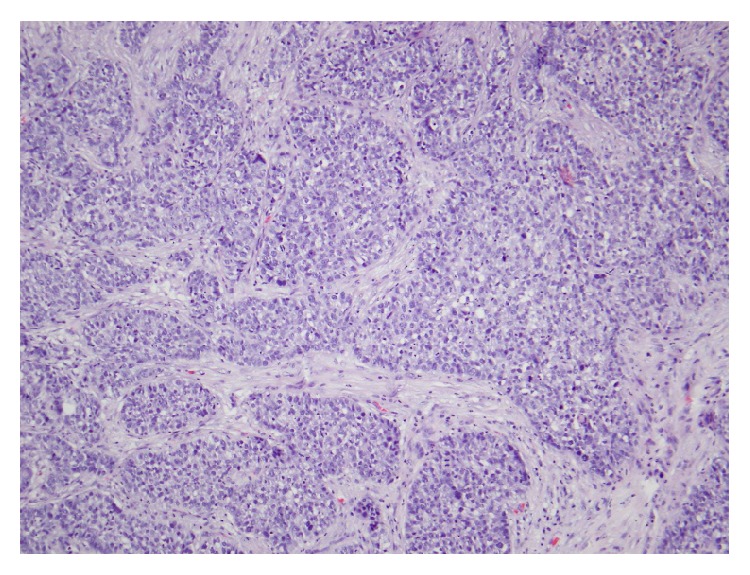
Poorly differentiated nonkeratinizing deeply infiltrating tumor with foci of duct formation. H&E original magnification ×200.

**Figure 3:**
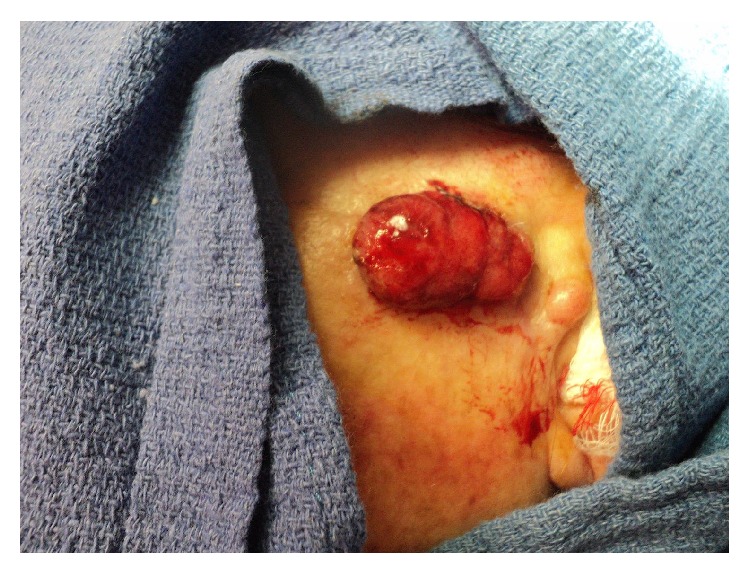
Recurrence of sebaceous carcinoma three months after Mohs resection.

**Figure 4:**
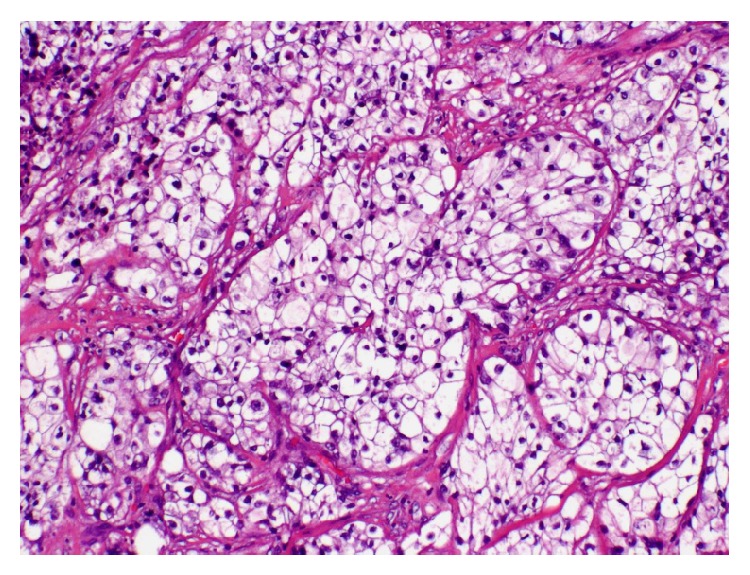
Tumor cells with clear cytoplasm with morphology and immunophenotype consistent with sebaceous carcinoma. H&E original magnification ×400.

The tumor was removed with MMS with negative microscopic margins determined by frozen section in one stage resulting in a 3.5 × 2.3 cm defect. Debulked specimen was sent for permanent section revealing a deeply infiltrating primarily undifferentiated carcinoma extending to the subcutaneous fat without evidence of keratinization but with a few foci of duct formation. The neoplasm was connected to and was continuous with the epidermis suggesting that it represents an undifferentiated squamous cell carcinoma (Figure 2). However, due to the presence of ducts, eccrine carcinoma was considered as a potential diagnosis. Three months after resection, the patient presented with a 3 cm ulcerated nodule at the site of the prior excision (Figure 3). The lesion had rapidly recurred within the incision line one month after surgery, causing pain in her jaw and neck. MMS was performed again with negative microscopic margins after four stages resulting in a 4.0 × 4.0 cm defect. The patient was then evaluated with a contrast neck CT for metastatic disease by otolaryngology, which revealed metastases to several lymph nodes and to the parotid gland. The patient underwent a left total parotidectomy, left radical neck dissection, and left anterolateral free flap reconstruction of the parotid defect. Examination of the tissue biopsy from the metastatic lesion revealed tumor cells with clear cytoplasm with morphology and immunophenotype consistent with sebaceous carcinoma (Figure 4). The tumors cells were positive for p63 and focal CD10, negative for renal cell carcinoma markers RCC and PAX-8, and negative for myoepithelial marker calponin, melanoma markers S100 and HMB-45, and D2-40. The patient underwent radiotherapy but passed away from complications of the metastatic disease three months later.

## 3. Discussion

Malignant adnexal tumors are rare neoplasms that are classified based on their differentiation which includes sebaceous, eccrine, apocrine, and follicular groups. However, these tumors often exhibit histological features of different cell lineages making them difficult to classify into a single group. Histological appearance alone may not be sufficient for diagnosis of adnexal tumors [3]. In the case described, different pathologists arrived at different diagnoses. It has been suggested that immunohistochemistry can help in the diagnosis of such neoplasms [4].

Sebaceous carcinomas are rare adnexal tumors with seventy-five percent of cases arising in the ocular region [5]. Twenty-five percent of the cases are extraocular with the most common location being the head and neck, reflecting a higher density of sebaceous glands [6]. SC usually arises in the seventh decade, although it has been documented to occur in younger individuals as well [7]. Risk factors include previous irradiation, Muir-Torre syndrome, and immunosuppression following renal transplantation [8].

Posttransplant immunosuppression has been implicated as a risk factor for developing SC. In addition, patients after organ transplantation tend to have a worse prognosis with more aggressive tumors [9]. The incidence of SC in posttransplant patients tends to be underestimated since many of these tumors are often misdiagnosed as squamous cell carcinomas with sebaceous differentiation [10]. One study proposes that immunosuppressive medications, most plausibly azathioprine, potentially select for the emergence of a mutated phenotype of cells with microsatellite instability due to mismatch repair defects leading to a higher probability of SC development [11].

SC is an aggressive neoplasm with possible nodal and distant metastases with recurrence after excision. Extraocular SC is associated with a 29% recurrence rate and 21% metastatic rate [8]. Five-year survival rate for SC has been reported as 68% and mortality ranges from 9% to 50% [8, 12]. Patients with extraocular SC may present with a pink, yellowish, or red nodule with occasional bleeding but the presentation may vary substantially and is not reliable for diagnosis [13].

Histologically, the tumor is dermal and nonencapsulated and composed of clear cells with various levels of differentiation. Occasional spread of the tumor into the epidermis is possible [14]. Histologically, the differential diagnosis is broad including poorly differentiated SCC, basal cell carcinoma with sebaceous differentiation, eccrine porocarcinoma, Paget's disease, metastatic adenocarcinoma, and other neoplasms with clear cells. To help distinguish the diagnosis immunohistochemistry can be used. SC is usually positive for EMA, BER-EP4, CA15.3, androgen receptor, and adipophilin [15, 16]. SC along with other adnexal neoplasms are positive for p63 helping to distinguish them from metastatic adenocarcinomas [4]. SC is usually positive for CD10, differentiating it from eccrine porocarcinoma and poorly differentiated SCC which are negative [17, 18].

Treatment has traditionally been with surgical excision with 5-6 mm wide margins with high recurrence rates of 36% and an 18% five-year mortality [12]. MMS has also been reported as an effective treatment modality with a cure rate of 87.8% [1]. A recent retrospective study compared MMS to wide local excision (WLE) showing 1 out of 35 recurrences in the MMS group and 1 out of 23 recurrences in the WLE group [2]. However, the participant subjects were too few and conclusions could not be drawn as to which modality is more efficacious.

We report a rare case of extraocular SC recurrence and metastasis after MMS excision. Our case illustrates that there can be confusion as to the definitive diagnosis of the neoplasm histologically. Immunohistochemical staining is helpful to determine the diagnosis and should be used when the diagnosis is in question. Traditionally, extraocular SC is considered less aggressive than ocular SC. However, in our case the tumor was aggressive, recurred, and metastasized a short time after MMS. The aggressive behavior of this particular SC may have been related to the patient's immunosuppressed status. We suggest that more research be conducted to evaluate the best options for diagnosis, risk factors, and optimal treatment of sebaceous carcinoma.
